# Evaluation of Turnaround Times for Antigen Testing in Hospitalized Patients With Histoplasmosis and Blastomycosis

**DOI:** 10.1093/ofid/ofae521

**Published:** 2024-09-14

**Authors:** Liam M Dalton, Carol A Kauffman, Blair Richards, Marisa H Miceli

**Affiliations:** Division of Infectious Diseases, Department of Internal Medicine, University of Michigan Medical School, Ann Arbor, Michigan, USA; Division of Infectious Diseases, Department of Internal Medicine, University of Michigan Medical School, Ann Arbor, Michigan, USA; Institute for Clinical and Health Research, University of Michigan, Ann Arbor, Michigan, USA; Division of Infectious Diseases, Department of Internal Medicine, University of Michigan Medical School, Ann Arbor, Michigan, USA

**Keywords:** blastomycosis, fungal antigen test, histoplasmosis, testing turnaround time, endemic mycoses

## Abstract

Review of histoplasmosis and blastomycosis antigen testing for 39 patients hospitalized with these diseases found that there were significantly longer turnaround times between the time of specimen collection and receipt of positive test results among those patients who had worse outcomes.

Histoplasmosis and blastomycosis, endemic mycoses especially common in the midwestern United States, are often included as diagnostic possibilities in persons presenting with pulmonary infection or with unexplained systemic illness. Immunocompromised patients are at special risk for disseminated, potentially life-threatening forms of these fungal infections [1]. Given the potential severity of these infections, prompt diagnosis, allowing initiation of appropriate antifungal agents, is paramount.

Testing urine and serum for the galactomannan antigen that occurs in the cell wall of these fungi using an enzyme immunoassay (EIA) has become a valuable procedure to rapidly establish the diagnosis of probable histoplasmosis or blastomycosis [2]. Antigen testing is performed in reference laboratories; thus, additional time to diagnosis is introduced because of the logistics of processing the sample and sending it to a distant site. Clinical observations suggest that this increased turnaround time (TAT) may lead to delays in the initiation of antifungal treatment and poorer outcomes. This has not been formally studied for the endemic mycoses although previous studies have found that these infections are among those most frequently associated with diagnostic delays [3]. We sought to evaluate whether longer TAT for antigen testing was noted in hospitalized persons with histoplasmosis and blastomycosis who had worse outcomes.

## METHODS

### Patients

This was a retrospective observational study of adult patients who were hospitalized at Michigan Medicine, a 1000-bed tertiary care hospital in southeastern Michigan, and who had a positive *Histoplasma* or *Blastomyces* urine or serum antigen EIA (Miravista Diagnostics, Indianapolis, Indiana) obtained from 1 January 2015 to 31 December 2022. Patients were identified using DataDirect, a clinical data repository at Michigan Medicine [4]. This study was approved by the University of Michigan Institutional Review Board; informed consent was deferred for this retrospective study.

Only patients in whom the antigen test was the modality used to confirm the probable diagnosis of histoplasmosis or blastomycosis were included; patients whose diagnosis was obtained by other methods, who were transferred from an outside hospital after diagnosis was confirmed, or who were treated empirically were excluded. Only patients who received antifungal therapy or who died before treatment could begin and were shown to have histoplasmosis or blastomycosis at autopsy were included. Severe pulmonary infection was defined as hypoxia with need for oxygen supplementation and/or mechanical ventilation. Severe disseminated infection was defined as hemodynamic instability, with or without hypoxia or central nervous system involvement.

### Data Collection

Medical records were reviewed to obtain demographic information, treatment, time from hospital admission to antigen sample collection, time from admission to antigen test result, and time from sample collection to antigen test result. Outcomes included intensive care unit (ICU) transfer, length of stay, hospital readmission within 30 days of discharge, and survival in the 60 days after diagnosis. All data were stored in a secure REDCap (Research Electronic Data Capture) database for further analysis [5].

### Definitions (Figure 1)

**Figure 1.**
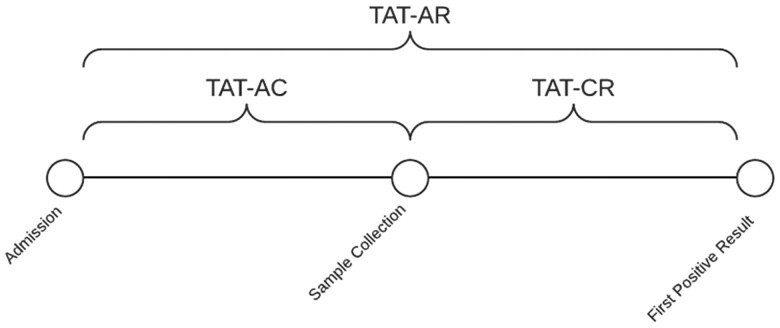
Definitions of turnaround times. Abbreviations: TAT-AC, turnaround time from admission to collection of specimen; TAT-AR, turnaround time from admission to result; TAT-CR, turnaround time from collection of specimen to result.

- Turnaround time from admission to collection (TAT-AC): interval between hospital admission and collection of a specimen that yielded the earliest report of a positive antigen result supporting the diagnosis of histoplasmosis or blastomycosis.
- Turnaround time from admission to result (TAT-AR): interval between hospital admission and the earliest report of a positive antigen result supporting the diagnosis of histoplasmosis or blastomycosis.
- Turnaround time from collection to result (TAT-CR): interval between collection of the antigen specimen and earliest report of a positive antigen result supporting the diagnosis of histoplasmosis or blastomycosis.

### Statistical Analysis

Wilcoxon rank-sum test was used to determine differences between surviving and deceased patients for TAT-AR, TAT-AC, and TAT-CR. Results are reported as median days (interquartile range [IQR]). Statistical significance was defined as *P* < .05; analyses were completed using SAS software, version 9.4 (SAS Institute, Cary, North Carolina) and R software, version 4.3.2 (R Foundation for Statistical Computing, Vienna, Austria).

## RESULTS

### Demographics

A total of 39 patients met the study criteria. Mean age was 47 ± 19 years, and 26 (67%) were men; 33 (85%) were immunocompromised, including 15 who had received a solid organ transplant and 7 taking tumor necrosis factor alpha inhibitors for autoimmune diseases. Thirty-four patients had histoplasmosis; 4 of 7 with pulmonary histoplasmosis had severe disease. All 27 patients with disseminated histoplasmosis had severe disease. Five patients had blastomycosis (1 pulmonary and 4 disseminated disease); 1 patient with disseminated blastomycosis had severe disease. Nine patients (23%) died within 60 days of diagnosis, including 8 with disseminated histoplasmosis and 1 with disseminated blastomycosis.

### Turnaround Times

For all 39 patients, the median days (IQR) for TAT-AC, TAT-AR, and TAT-CR were 1 (1–2.5), 6 (4–7.5), and 4 (3–5), respectively. When 30 surviving patients were compared with 9 patients who died, TAT-AC was not significantly different. Both TAT-AR and TAT-CR were significantly less for the 30 survivors compared with the 9 patients who died (*P* = .03 and *P* = .005, respectively; Table 1).

**Table 1.** Association of Turnaround Times for Antigen Testing With Survival at 60 Days After the Diagnosis of Histoplasmosis or Blastomycosis in Hospitalized Patients

Cohort	Measure	Survived (n = 30)	Died (n = 9)	*P* Value
All patients (N = 39)	TAT-AC	1 (1–2)	1 (1–12)	.35
	TAT-AR	5 (4–7)	7 (5–17)	.03
	TAT-CR	4 (3–5)	5 (4–7)	.005
Patients with severe disease (n = 32)		Survived (n = 23)	Died (n = 9)	
	TAT-AC	1 (1–2)	1 (1–12)	.48
	TAT-AR	5 (4–7)	7 (5–17)	.03
	TAT-CR	4 (3–5)	5 (4–7)	.004

Data are presented as median days (interquartile range).

Abbreviations: TAT-AC, turnaround time from admission to collection of specimen; TAT-AR, turnaround time from admission to result; TAT-CR, turnaround time from collection of specimen to result.

Among 32 patients who had severe disease and who required intravenous amphotericin B (n = 29) or who died before any antifungal therapy was given (n = 3), TAT-AC for survivors and deceased patients did not differ. Both TAT-AR and TAT-CR were significantly less for survivors than for those who died (*P* = .03 and *P* = .004, respectively; Table 1).

Comparing 8 patients who had only pulmonary disease with 31 patients who had disseminated disease, no significant differences were found in TAT-AC (*P* = .56), TAT-AR (*P* = .44), or TAT-CR (*P* = .16). ICU transfer, length of stay, and 30-day readmission were not significantly associated with TAT-AC, TAT-AR, or TAT-CR in the overall cohort or the severe disease cohort.

## DISCUSSION

Antigen test TAT represents a single facet of the overall process of diagnosis of histoplasmosis and blastomycosis. Studies focusing on histoplasmosis have found that patients often experience multiple missed opportunities for diagnosis. Patients frequently see providers in the weeks prior to their diagnosis, but at these visits the endemic mycoses are rarely considered [6, 7]. The most frequently cited reasons for delayed diagnosis are failure to consider endemic mycoses due to their rarity and similar presentation to other diseases [3]. One study estimated that 82.9% of patients experienced at least 1 delay in diagnosis [7]. Our study demonstrates that even after the diagnosis is suspected, there remain obstacles to the timely establishment of the diagnosis, which may have an impact on patient outcomes.

Our study shows that delays in receiving results of a positive antigen test were associated with increased mortality in hospitalized patients requiring antifungal treatment for histoplasmosis or blastomycosis. These findings highlight the importance of rapid notification of test results to guide treatment for hospitalized patients with these diseases. It should be noted that this association does not necessarily imply a causal relationship between TAT and poor outcomes. Increased TAT could be associated with other logistical factors affecting the delivery of patient care, such as staffing shortages, for instance, which could themselves contribute to poor outcomes.

Considering these findings, providers should work closely with service units that process clinical specimens, as well as with designated reference laboratories to ensure that TAT is as short as possible. Potential changes to specimen processing could include ensuring different handling of antigen tests ordered in the inpatient and outpatient setting. TAT in the former can be a rate-limiting step in initiating care for ill patients, whereas outpatient testing can typically accommodate longer TAT without concern for adverse patient outcomes. Integrating electronic medical record systems directly with the notification systems of reference laboratories also could improve TAT. Reviewing the logistics of send-out tests during fringe times, such as late at night, weekends, and holidays, may help to identify potential areas for improvement. At many institutions, including ours, certain specialized tests are first sent to an outside institution before being routed to the specific laboratory performing the assay, introducing additional time before sample processing; eliminating this step would decrease TAT. In recent years, rapid point-of-care tests utilizing lateral flow techniques for the diagnosis of histoplasmosis have been developed and tested in resource-limited settings [8–10]. More studies are needed to determine their role in the diagnosis of histoplasmosis in different settings and populations. If proved to be as sensitive as the current EIA, these tests would obviate the issues we noted with TAT.

Limitations of our study are that it is from a single center, is a retrospective review, and sample size is small. Further study with larger sample sizes could investigate relationships that our study was underpowered to capture. Because logistical factors that determine how samples are processed likely differ from institution to institution, further study at other institutions could provide insights into disparate reasons for increased TAT. Many factors contribute to the mortality of patients hospitalized with histoplasmosis and blastomycosis [1]; this study was not designed to evaluate these factors, but rather focused only on comparing TAT in patients with different outcomes.

Clinicians should maintain a high index of suspicion for histoplasmosis and blastomycoses to avoid delays in diagnosis, but also must consider logistical factors at their institution that may impact their ability to provide care for their patients. Raising awareness of the importance of expeditious specimen shipping and prompt reporting of *Histoplasma* and *Blastomyces* antigen testing may help to optimize patient care.

## Note

***Potential conflicts of interest*.** M. H. M. has received consulting fees from Scynexis. All other authors report no potential conflicts.
